# A novel gene selection method for gene expression data for the task of cancer type classification

**DOI:** 10.1186/s13062-020-00290-3

**Published:** 2021-02-08

**Authors:** N. Özlem ÖZCAN ŞİMŞEK, Arzucan ÖZGÜR, Fikret GÜRGEN

**Affiliations:** Department of Computer Engineering, Bogazici University, Bebek, İstanbul, Turkey

**Keywords:** Disease classification, Cancer research, Gene expression, DNA mutations, Gene weighting, Information retrieval, Machine learning

## Abstract

Cancer is a poligenetic disease with each cancer type having a different mutation profile. Genomic data can be utilized to detect these profiles and to diagnose and differentiate cancer types. Variant calling provide mutation information. Gene expression data reveal the altered cell behaviour. The combination of the mutation and expression information can lead to accurate discrimination of different cancer types. In this study, we utilized and transferred the information of existing mutations for a novel gene selection method for gene expression data. We tested the proposed method in order to diagnose and differentiate cancer types. It is a disease specific method as both the mutations and expressions are filtered according to the selected cancer types. Our experiment results show that the proposed gene selection method leads to similar or improved performance metrics compared to classical feature selection methods and curated gene sets.

## Background

Cancer is among the leading causes of death worldwide [1]. It is a group of diseases and each cancer type is labeled by the primary area of the body where the cancer cells arise. A different set of causal genes leads to each cancer type and the disease emerges from the combination of various mutations of these genes [1]. The cancer treatment is planned according to the driving mutations. The unknown or wrong analysis of these mutations lead to incorrect treatments and this is one of the major problems for cancer patients. Genomic data can be utilized for diagnosis of the disease and for recognizing different types. Genomic tests reveal the gene mutations that may be driving a cancer’s behavior. This information helps doctors while deciding on the patient’s personal treatment [2].

Driving mutations are located by detailed analysis on genomic data. Whole genome sequences and variant calling are utilized for mutation analysis [3–5]. Both coding and non-coding regions of the DNA are analyzed for the discovery of mutational signatures of cancer types.

Besides comprehensive statistical analysis, machine learning algorithms may help to detect the driving mutations. A commonly used data type for cancer classification is gene expression data. A number of studies have utilized gene expression data and addressed the classification of cancer types [6–10]. A major challenge of using gene expression data is the small sample size with high dimensionality. There may be thousands of genes in each sample but only a few of them are effective on the target disease, and most of them are irrelevant [11]. Gene selection methods are commonly applied prior to classification to overcome the high dimensionality problem [12, 13]. Hovewer, the feature selection step may eliminate genes that in general have minor effects on disease generation while still being significant for the diagnosis of particular cancer types for some patients. In addition, the irrelevant genes add noise and reduce the classifier performance for machine learning approaches [14, 15].

In this study, we propose a novel gene selection method targeting gene expression data for the task of cancer type classification. In a previous study [16], we utilized the mutation information in variant call format (VCF) [17] files. The most effective genes in the discrimination of cancer types are identified. In this study, these most effective genes within VCF data are employed for gene selection on gene expression data. The proposed method is compared to computational based and manually curated gene signature lists. The most important aspect of this method is that every step is disease specific and can be adapted to any genomic disease.

Our work brings the following contributions:

1. A novel and disease/trait specific gene selection method is proposed and tested.
2. The valuable information in DNA mutations are transferred and used with gene expression data.
3. Similar and slightly improved classification results are achieved compared to computational based and manually curated methods.
4. This system can be applied to any genomic disease or trait.

## Methods

### Dataset

We utilized the gene expression files in FPKM (fragments per kilobase million) format and the VCF files for the samples listed in CAMDA 2019 Hi-Res Cancer Data Integration Challenge [18]. All files are downloaded from The Cancer Genome Atlas Project (TCGA) [19]. The challenge includes samples for three cancer types: Breast, Lung Adenocarcinoma (Lung) and Kidney Renal Clear Cell Carcinoma (Kidney). We selected samples both having FPKM and VCF files. The list of cancer types and the sample counts for each cancer type are provided in Table 1.

**Table 1:** The list of cancer types and sample counts in our dataset

Cancer Type	Sample count
Breast	1020
Lung	507
Kidney	330

### Feature selection for gene expression

For baseline, we used the whole gene list in gene expression files. There are 60,483 distinct genes in the whole gene expression set. For feature selection, we applied SelectKBest from scikit-learn library [20], minimum Redundancy Maximum Relevance (mRMR) with pymrmr library [21] and Relief with Weka [22, 23]. SelectKBest is a feature selection method which selects features according to the highest scores of a selected scoring function. mutual_info_classif from scikit-learn library is used as scoring function. It scores features according to their mutual dependencies. mRMR is a feature selection method which chooses a feature subset by considering a trade-off between relevance and redundancy. Relief is a feature selection method which returns a feature subset based on relevance. We applied these methods with different numbers of features; 1,000, 5,000 and 10,000 for SelectKBest and Relief and 1,000 and 5,000 for mRMR.

Another feature selection method is to use a gene signature list. The Hallmark gene set collection [24] is generated by a hybrid approach that combines an automated computational procedure with manual expert curation. It consists of multiple gene sets and displays the discriminating behavior across a number of test datasets. In order to benefit information from all of the source datasets, we used the union of all Hallmark gene sets as feature list. This results in 4,266 gene features.

The last feature selection method of this study is the one that we propose. In our previous study [16], we employed VCF files for cancer type classification. An impressive output of that study was the list of most effective genes in decision making. Most of these genes were found to be proposed as target genes in the literature. For a novel feature selection method, we combined the most effective genes from our previous study for the three cancer types. We selected 3,000 and 3,500 most effective genes for each cancer type and combine them to curate a feature set that represents all three cancer types. For the 3,000 most effective genes, the final gene list has 6,752 genes. For the 3,500 most effective genes, the final gene list has 7,741 genes.

### Implementation of machine learning methods and experiment design

All experiments are implemented with Python and Weka. For the machine learning algorithms, the scikit-learn and pymrmr libraries are used. We applied Logistic Regression (LR) on the curated datasets. Each test is applied with 5-fold cross validation. The reported results are the micro-averaged scores and standard deviations on the applied cross-validation folds. Accuracy, f-score, false positive rate (FPR), area under the receiver operating curve (roc-auc) and Matthews correlation coefficient (MCC) are used as the performance measures.

## Results and discussion

### Comparison with a previous study

For baseline, the whole gene set in FPKM files are used in classification task. In order to compare with another data type, we utilized our previous study that operates on VCF data in order to classify cancer types. We applied and compared a number of statistical representation methods in that study. The best performing representation method was BM25-tf-rf. Therefore, we applied this model for this problem. The LR experiment results for FPKM and VCF datasets are presented in Table 2.

**Table 2:** Machine learning experiment test results of gene expression and VCF data

Dataset	Feature count	Accuracy	F-Score	Roc-Auc	FPR	MCC
FPKM all features	60483	99.46 ±0.42	99.46 ±0.42	99.45 ±0.50	0.40 ±0.31	99.09 ±0.71
VCF BM25-tf-rf	16383	93.70 ±1.02	93.62 ±1.07	93.26 ±1.27	3.60 ±0.71	89.31 ±1.76

The number of features in FPKM dataset is four times that of VCF dataset. Despite the extra cost these features cause for the classification model, the performance metrics are improved with this dataset. The accuracy result for FPKM dataset is 99.46*%* whereas it is 93.70*%* for VCF dataset. The f-score result for FPKM dataset is also 99.46*%* whereas it is 93.62*%* for VCF dataset. When we consider FPR and MCC results, the difference between two datasets are more clearly observed. The FPR value for FPKM dataset is 0.40*%* whereas it is 3.60*%* for VCF dataset. The MCC result for FPKM dataset is 99.09*%* whereas it is 89.31*%* for VCF dataset. According to these results, we will utilize FPKM files for further experiments.

### Gene selection results

The use of all genes leads to good results in classification task. But it also increases the computational cost. Therefore, we applied a number of gene selection methods in order to create more FPKM based datasets. The LR experiment results using these datasets are presented in Table 3.

**Table 3:** Machine learning experiment test results of gene expression data

Feature Sel.	Feature count	Accuracy	F-Score	Roc-Auc	FPR	MCC
FPKM all features	60483	99.46 ±0.42	99.46 ±0.42	99.45 ±0.50	0.40 ±0.31	99.09 ±0.71
SelectKBest	10000	99.57 ±0.13	99.57 ±0.13	99.61 ±0.18	0.29 ±0.13	99.27 ±0.22
SelectKBest	5000	99.30 ±0.27	99.30 ±0.27	99.47 ±0.22	0.46 ±0.24	98.82 ±0.46
SelectKBest	1000	98.92 ±0.38	98.92 ±0.38	99.13 ±0.41	0.71 ±0.32	98.18 ±0.64
mRMR	5000	98.98 ±0.43	98.98 ±0.43	99.08 ±0.33	0.73 ±0.23	98.28 ±0.72
mRMR	1000	98.44 ±0.52	98.45 ±0.52	98.63 ±0.56	0.82 ±0.32	97.37 ±0.88
Relief	10000	98.44 ±0.31	98.45 ±0.31	98.51 ±0.45	0.82 ±0.28	97.37 ±0.53
Relief	5000	99.30 ±0.36	99.30 ±0.36	99.34 ±0.37	0.27 ±0.14	99.34 ±0.37
Relief	1000	99.46 ±0.17	99.46 ±0.17	99.54 ±0.16	0.39 ±0.14	99.09 ±0.29
Hallmark	4266	99.57 ±0.22	99.57 ±0.21	99.57 ±0.23	0.29 ±0.14	99.27 ±0.36
VCF 3000 Effective Genes	6752	99.57 ±0.47	99.57 ±0.47	99.64 ±0.37	0.31 ±0.34	99.27 ±0.79
VCF 3500 Effective Genes	7741	99.68 ±0.40	99.68 ±0.39	99.72 ±0.30	0.24 ±0.26	99.46 ±0.67

When we applied SelectKBest with 10,000 features, the accuracy and f-score values increase slightly to 99.57*%* compared to all features. But when we applied the same feature selection method with less features, the classification performance decreases as the number of features decreases. mRMR algorithm produces less accurate results with accuracy and f-score values as 98.98*%* with 5,000 features. With an opposite tendency compared to SelectKBest, Relief leads to improved classification performance with less features. Accuracy and f-score values as 99.46*%* are achieved with Relief method with 1,000 features. When we compare these three feature selection algorithms, SelectKBest with 10,000 features outperforms other two methods.

We employed another feature selection method in order to reduce the feature count even more. When we consider a combination of all Hallmark gene sets for our feature list, the experiment results show that a similar performance can be achieved with SelectKBest with 10,000. But the number of features is less than the half.

Hallmark gene sets are known and used for years now. They depend on the previously curated gene sets. We further attempt to create a gene selection method that only depends on the data itself. Therefore, we selected most effective genes for the three cancer types from our previous study which employs VCF files. By this method, the information hidden in mutations are transferred to gene expression data. When we selected 3,000 genes for each cancer type and utilized the union of them, the resulting dataset leads to similar performance in classification with the Hallmark gene set. When we selected 3,500 genes for each cancer type and used the union of them, there is a slight improvement in the performance results. The resulting accuracy and f-score is 99.68*%* and MCC value is 99.46*%*. The FPR also decreases slightly to 0.24*%*. According to these results, the proposed VCF based gene selection method leads to similar or improved performance as the number of most effective genes is adjusted. As the disease is caused by mutations in DNA, it is reasonable to use these mutations in order to select effective genes and further analyze their expression levels. Our experiment results support this idea.

**Fig. 1:**
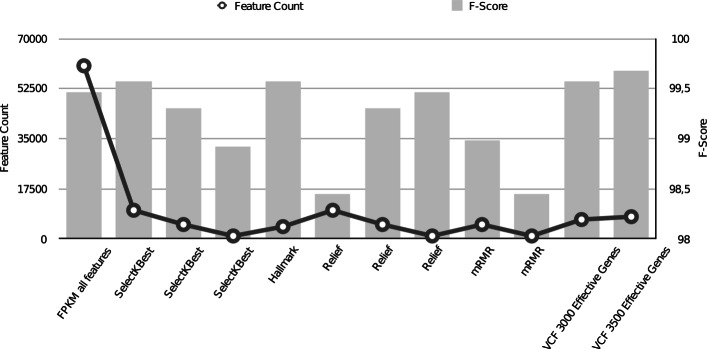
F-score and feature count comparison of experiment results

The comparison of f-score values and feature counts can be observed in more detail in Fig. 1. The most effective methods can be taken as the ones with f-score values above 99.5*%*. These are SelectKBest with 10,000 features, Hallmark and VCF based methods. Although VCF based gene selection method with 3,500 genes doesn’t provide the least feature count, it produces a slight improvement in f-score compared to the most successful methods in this study.

## Conclusion

Changes in DNA alter cell behavior and cause genomic diseases. Each genomic disease occurs in consequence of a different mutation profile. Besides the existence of a gene mutation, its effect can also be analyzed by the expression levels of genes. In this study, we provide the union of the existence and expression level information of mutated genes and propose a novel gene selection method. We utilized the mutation information in DNA for the selection of relevant genes in the gene expression data.

Based on our previous study [16], we selected gene features in expression data with the help of the most effective gene mutations for each cancer type. By this method, the valuable information in variant calling files are transferred and used with a different genomic data type. Although the number of samples is very limited in this study, this novel gene selection method leads to similar and slightly improved classification results compared to classic feature selection methods as SelectKBest, mRMR, Relief and curated gene sets as Hallmark. The proposed feature selection method is specific to the target disease as the effective genes are decided accordingly. Therefore, this system can be adapted and applied to any genomic disease or trait.

## Data Availability

The data used in this study is provided by CAMDA 2019 Hi-Res Cancer Data Integration Challenge [18].

## Abbreviations

BM25-tf-rf: An input representation model
CAMDA: Critical assesment of massive data analysis
FPKM: Fragments per kilobase million
FPR: False positive rate
Hallmark: The molecular signatures database hallmark gene set collection
LR: Logistic regression
MCC: Matthews correlation coefficient
mRMR: Minimum redundancy maximum relevance
Relief: A feature selection method
roc-auc: Area under the receiver operating curve
SelectKBest: A feature selection method from phyton scikit-learn library
TCGA: The cancer genome atlas
VCF: Variant call format
Weka: A machine learning tool
